# A computational pipeline to generate MHC binding motifs

**DOI:** 10.4172/1745-7580.1000046

**Published:** 2011-05

**Authors:** Peng Wang, John Sidney, Alessandro Sette, Bjoern Peters

**Affiliations:** 1 La Jolla Institute for Allergy & Immunology, 9420 Athena Circle, La Jolla, CA 92037, USA

## Abstract

**Background:**

Major histocompatibility complex (MHC) class I molecules play key roles in host immunity against pathogens by presenting peptide antigens to CD8+ T-cells. Many variants of MHC molecules exist, and each has a unique preference for certain peptide ligands. Both experimental approaches and computational algorithms have been utilized to analyze these peptide MHC binding characteristics. Traditionally, MHC binding specificities have been described in terms of binding motifs. Such motifs classify certain peptide positions as primary and secondary anchors according to their impact on binding, and they list the preferred and deleterious residues at these positions. This provides a concise and easily communicatable summary of MHC binding specificities. However, so far there has been no algorithm to generate such binding motifs in an automated and uniform fashion.

**Results:**

In this paper, we present a computational pipeline that takes peptide MHC binding data as input and produces a concise MHC binding motif. We tested our pipeline on a set of 18 MHC class I molecules and showed that the derived motifs are consistent with historic expert assignments.

**Conclusions:**

We have implemented a pipeline that formally codifies rules to generate MHC binding motifs. The pipeline has been incorporated into the immune epitope database and analysis resource (IEDB) and motifs can be visualized while browsing MHC alleles in the IEDB.

## Background

A central process in host immunity against pathogenic antigens is the presentation of peptide ligands by MHC class I molecules to CD8+ cytotoxic T-cells [1]. The MHC class I molecule consists of two chains, a heavy chain with three domains (α_1_, α_2_ and α_3_) and a small beta-2 microglobulin unit [2]. Peptides are presented in a groove formed between α_1_ and α_2_ domains. The MHC class I binding groove is in a closed conformation since both ends of the groove are blocked by large aromatic residues. This limits the length of the presented peptides to 8–10 amino acids. Despite the relatively limited length, a single MHC molecule is capable of presenting peptides of tremendous diversity [3] and discovering novel peptide ligands remains a challenging task.

A hallmark of the interaction between peptide ligands and MHC class I molecule is the existence of anchors. The anchor residues, typically found at position 2 and the and C-terminus of a peptide, form extensive networks of hydrogen bonds with MHC class I molecule and contribute the most to binding energy [4]. A large number of experimental studies have been carried out to determine anchor positions and their associated residue preference for MHC class I molecules. Those studies revealed significant diversity in the number of anchors, anchor position as well as the residue preference among different MHC class I molecules.

A large number of bioinformatics studies have been carried out to analyze MHC peptide binding and develop algorithms to predict high affinity binders [5–10]. While the derived machine learning approaches can exhibit good performance for prediction, they do not provide an easily communicable summary of the binding specificity of an MHC allele, which is desired by experimentalists. While logo based approaches are popular to display transcription factor binding motifs or conserved protein domains [11–13], they are less useful to describe MHC binding motifs since they don’t explicitly display anchor information and residue preferences. In practice, experimentalists have for decades described the MHC class I binding in terms of anchors and key residues critical for favorable binding. There is therefore a need to supply such a summary of binding characteristics.

In this study, we report our implementation of an automatic computational pipeline to display MHC class I binding motifs in an experimentalist friendly fashion. We start by generating scoring matrices from binding data via the stabilized matrix method (SMM) [7, 14]. The SMM scoring matrices were then analyzed to design a computational algorithm that identifies anchor residues and determines residue preference. We have tested the resulting method on a set of 18 MHC class I molecules and compared the results to previously published reports of binding motifs as well as those contained in the SYFPEITHI database [15]. Those comparisons showed that our methods are effective in automatically determining anchor positions and residues preferences in agreement with historic manual assignments. Finally, we have implemented the pipeline into IEDB [16], which now provides automatically updated motifs based on the accumulated binding data stored in the database.

## RESULTS

### The MHC class I binding affinity dataset for deriving motif display algorithms

The IEDB is a comprehensive resource of immune epitopes and currently stores results from over 160,000 peptide:MHC binding affinities. For this study, we extracted binding affinities for 18 human and mouse MHC class I molecules from the IEDB. These MHC class I molecules were selected based on a previous study in which the anchor positions and residue preferences were assigned directly from experimental data by a human expert [17], which serves as a gold standard. The retrieved peptide binding data for the alleles utilized are summarized in Table 1. On average, each MHC class I molecule dataset comprised 776 total data points and 279 binders. The HLA-A*0201 molecule had the highest number of data points (3,319) as well as binders (1,392) confirming its status as the best studied MHC class I molecules. The H-2-Kk molecule had the smallest number of total data points (164) and the H-2-Dd molecule had the smallest number of binders (13).

### Establishing Algorithms to automatically identify anchor positions and residue preference

We started by learning scoring matrices from binding data utilizing the SMM approach. We choose SMM since it is one the best performing matrix methods available, and because matrix methods provide a readily interpretable input to assess the relative importance of positions and residues in a peptide for binding. The SMM matrices were then analyzed to calculate spread factors (SF) for each column, defined as the difference between the highest and lowest matrix value. For a given peptide position, the SF values can be interpreted as evaluating the difference for binding on a log10(IC50) scale between having the best or worst amino acid residue at that position.

We utilized the SF values as input to an algorithm to determine anchor positions in a peptide. We heuristically optimized the algorithm, resulting in the procedure outlined in Figure 1a as a flow chart. The steps in this procedure were chosen to maximize the congruence with past manual assignments of anchor positions for the set of 18 MHC class I molecules identified in the gold standard previous study [17]. A confusion matrix comparing the different assignments is shown in table 2. Our automatic method achieved 96.7% specificity and 82.1% sensitivity suggesting that it is highly effective in reproducing anchor- and non-anchor positions assignments made by experts.

After the anchor positions were identified, two separate algorithms were applied to determine residue preference for anchor positions (Figure 1b) and non-anchor positions (Figure 1c). For anchor positions, the maximum entry in each column was set as reference. The other values in the same column were then compared to the reference to determine residue preference for this position. For non-anchor positions, the median value in each column was set as reference and the residue preference was determined following the procedure described in the flow chart.

We again optimized these algorithms to maximize the agreement of the algorithmically determined preference pattern with that identified in the gold standard expert assignments (Table 3a). From the data shown in Table 3a, it is clear that the residue preference determined by our automatic approach agrees well with those determined by domain experts. For example, for HLA-A*0201 our approach identified L and M as preferred residue for anchor position 2 and L and V for anchor position 9. In comparison, the expert assignments based on combinatorial library method designed L as preferred residues for anchor position 2 and V and I as preferred residues for anchor position 9, and the expert assignments based on pool sequencing motifs approach identified L and M as preferred residues for anchor position 2 and V and L as preferred residues for anchor position 9.

Having optimized the algorithm, we tested its performance on an independent dataset, namely the MHC binding motifs stored in the SYFPEITHI database (Table 3b). Our results showed overall consistency with information stored in SYFPEITHI. There were two noticeable differences in anchor positions. For HLA-A*3001, our algorithm designed position 3 as anchor while SYFPEITHI designed position 2 as anchor. For HLA-B*0801, our automatic algorithm designed three anchors (position 5, 6 and 9) while SYFPEITHI designed position 3 as anchor. For the remaining 13 alleles for which anchor assignments were made by both approaches, the positions are either identical between the two methods or the positions identified by one method are a subset of the other. Overall this demonstrates that the anchor positions identified by our algorithm are largely in agreement with the assignments made in the SYFPEITHI database.

### Extend motif display algorithm to multiple lengths for MHC class I alleles

While the lengths of MHC class I binding peptides are much more restricted compared to MHC class II molecules, MHC class I molecules do bind epitopes of different lengths. Therefore, it is necessary to develop several motifs of peptides with different lengths for the same MHC molecule. For example, a large number of epitopes of length 8 as well as 9 have been reported for the mouse MHC class I molecule H2-Kb [15, 18–20], so both lengths have to be considered when making motifs. At the same time, it is not meaningful to calculate binding motifs for peptide lengths that an MHC allele is unlikely to bind in the first place. For example, there are no known 8-mer T cell epitopes for HLA-A*26 according to either the IEDB [16] or SYFPEITHI [15]. In order to identify what peptide lengths are meaningful to include for each MHC allele in our motif display algorithms, we implemented a simple cutoff based approach. For each allele, we first selected the motif length with the highest number of known binders and designate this length as the default motif. Additional motif lengths were considered to be viable if there are 200 or more binders contained in the IEDB. After a motif length is selected, standard motif display algorithm describe in previous section were employed to generate motif of desired length.

### Integrate automatic motif display into IEDB

We have developed a set of PYTHON scripts to implement the motif length selection algorithm, automatic anchor and residue preference identification algorithms and display the resulting motifs. Those programs have been integrated into the IEDB and provide users with a visual presentation of MHC class I binding motifs. Two types of motif displays have been implemented. The first approach is a concise display of the classical binding motif (Figure 2a). This approach doesn’t provide quantitative information. Instead, it includes the key characteristics of binding specificity and provides a simple, visual presentation of the underlying motif. For alleles where motifs of different lengths are available, a tab based system was utilized and the most preferable motif was marked with “*”. In addition, a colored matrix (Figure 2b) approach was also provided. The colored matrix provided detailed information of the binding specificity matrix learned from SMM algorithm. The contribution of each residue to binding at different positions is represented by a float number that provides a quantitative measurement. In order to facilitate visualization, residues are colored according to their contribution to binding, and using a coloring scheme accessible to the color blind. These motif displays are now integrated into the MHC allele summary pages (see for example http://iedb.org/MHCalleleId/122) of the immune epitope database which are easiest to access through the ‘browse by MHC allele’ interface (http://iedb.org/bb_allele.php).

## DISCUSSION

In bioinformatics in general, sequence motifs are a popular approach to describe conservation among e.g. protein domains or transcription factor binding sites. Several programs have been developed to display such motifs as logos where bigger letters corresponding to higher level of conservation. Since such traditional motifs are derived from alignments of conserved sequences, extending them to MHC binding motifs has limited use, as they will only contain information about residues that are favorable for binding at each position. Since deleterious residues provide vital information regarding the binding characteristics of a MHC molecule, an alternative approach has been developed which displays a traditional logo with two parts denoting positive and negative contributions separately [21]. In our approach, quantitative binding data (binders and non-binders with binding affinities) were analyzed using the SMM algorithm to generate the initial scoring matrix from which the motif is derived. Both favorable and deleterious residues were then displayed in a single motif.

Our approach was aimed at providing a representation of MHC binding motifs in terms of anchor residues and residue preference as it would have classically been created by an experimentalist. Such a cartoon like approach provides a concise summary of the motif which is particularly easy to communicate. At the same time, we wanted to take advantage of the ability to create these motifs in an automated fashion based on binding data accumulated in the IEDB. This also for the first time provided a rigorous definition of what it means e.g. for a position to be an ‘anchor’.

In our algorithm to identify anchor positions, we utilized the fold difference between highest and lowest affinity of any residue at a given position as a straight forward way to assess the impact of a position on binding. This outperformed other similar metrics, such as the standard deviation of residue affinities, both in terms of predictive performance and simplicity. The cutoffs used in our algorithms were determined via systematic testing of a wide range of parameters and comparing the results against a benchmark set of expert defined binding motifs. After finalizing the algorithm, we compared its motifs with those stored in the SYFPEITHI database, and found an overall high agreement. As perfect agreement on a non-quantitative concept such as motifs is impossible to achieve, we are very satisfied with the achieved performance. A potential weakness is that we could only utilize a limited set of alleles to empirically tune parameters. It is possible that our parameters will not be universally applicable to other alleles and motif construction and visualization in general. We will therefore monitor how well the calculated motifs agree with publications over time, and if necessary adjust the heuristic parameters.

An important strength of our automatic motif display pipeline is to allow the side-by-side comparison of different length motifs for the same allele. This capacity allows user to easily carry out further analysis of binding motifs and identify insightful trend in binding. For example, two motifs (length 8 and 9) for H2-Kb allele were displayed by the automatic pipeline on IEDB website (http://iedb.org/MHCalleleId/122). Position 3 was identified as anchor in both motifs and the residue preference largely remained the same. This suggested that position 3 is critical for binding regardless of epitope length. On the other hand, the main anchor at position 5 for motif of length 8 became a non-anchor position for motif of length 9 and the c-terminal residue now became main anchor position for motif of length 9. Such changes suggest that as epitope length increases, the middle potion of the epitope has to adopt a more bulged conformation to fit the longer peptide into MHC class I binding groove which caused position 9 to replace position 5 as main anchor.

A natural extension of our automatic pipeline is to display binding motifs for MHC class II molecules. A key difference between MHC class II molecules and MHC class I molecules is that the peptide binding groove of MHC class II molecule is open at both end which allows the presentation of peptides of variable length. As a consequence, motif identification for MHC class II molecules is noticeably more difficult than that of MHC lass I molecules. Our initial attempts at creating automated algorithms to deduce MHC class II binding motifs that agree with published data from experimentalists were not successful. We plan to further work in this area, and test if newly improved MHC class II binding prediction approaches [22] will improve the ability to establish such algorithms.

## CONCLUSIONS

In this study, we have presented an automatic pipeline to display MHC class I binding motifs. We took a heuristic approach to generate binding motifs and have achieved largely consistent results with manually curated motifs by domain experts. The fully automatic, PYTHON script based pipeline allows the binding motifs to be easily updated or extended to new alleles as we accumulate more binding data. Comparing to logo based approach, our method combined the strength of easy to understand classical motif with an information rich binding matrix. In addition, the capacity to display multiple motifs for peptides of different lengths binding to the same allele side-by-side allows users to quickly identify key properties of epitope binding. Finally, we have implemented the automatic motif display pipeline into IEDB to better serve to the Immunology as well as Bioinformatics research community.

## METHODS

### Stabilized matrix method to derive scoring matrix

The stabilized matrix method (SMM) has been described in detail previously [7]. Briefly, each amino acid was encoded as a binary vector of length 20, with zeros at all positions except the one coding for the specific amino acid. Using such notion, a peptide of length N can be encoded as an N*20 binary vector. For a set of peptides, the vector representing each peptide can be stacked up to generate a matrix H where each row corresponds to a peptide. The best scoring matrix W can then be derived by minimizing the difference between the predicted binding affinities (HW) and the measured binding affinities y_meas_ while suppressing the effects of the noise in experiments with a regularization term W^t^ΛW where Λ is a positive scalar or a diagonal matrix with positive entries.

### Computational programs to determine viable motif lengths

The algorithms to viable motif lengths were implemented in a PYTHON script. The script will read in binding data statistics from IEDB and count the number of known binders for each allele. Motif lengths were recorded as valid if there are more than 200 known binders of that length.

### Computational programs to display binding motif cartoon and colored scoring matrix on IEDB website Sub- heading for this section

The algorithms to determine anchor position and residue preference were implemented in PYTHON as a cgi script. The script will read in SMM scoring matrixes stored on the IEDB analysis resource server and automatically generate HTML code to display colored SMM scoring matrices and JPEG files for the cartoon like motifs display.

## Figures and Tables

**Figure 1a:**
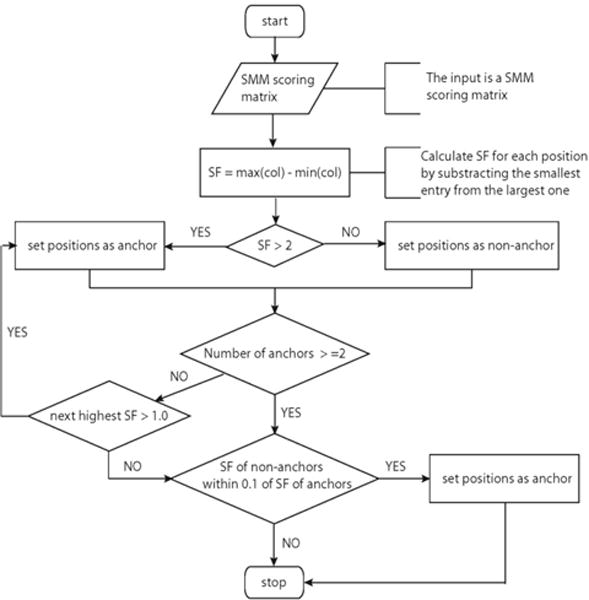
Flow chart showing the algorithm to determine anchor positions

**Figure 1b:**
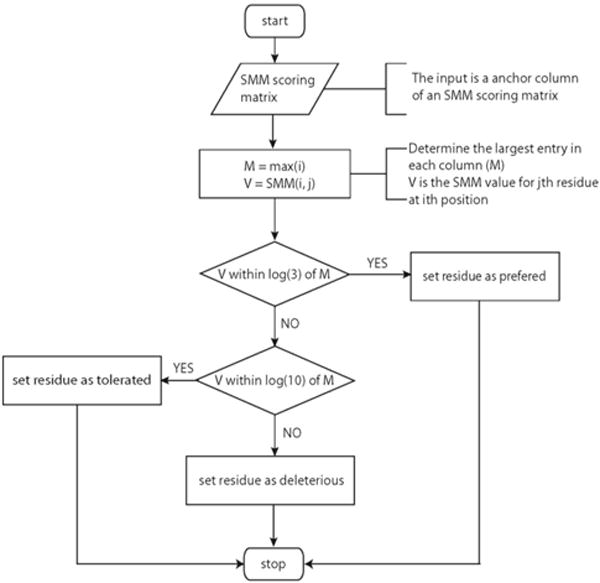
Flow chart showing the algorithm determining the residue preference for anchor positions

**Figure 1c:**
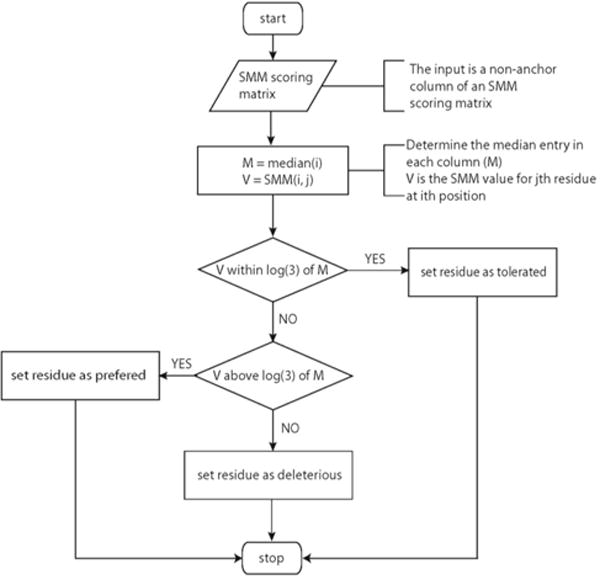
Flow chart showing the algorithm determining the residue preference for non-anchor positions

**Figure 2a:**
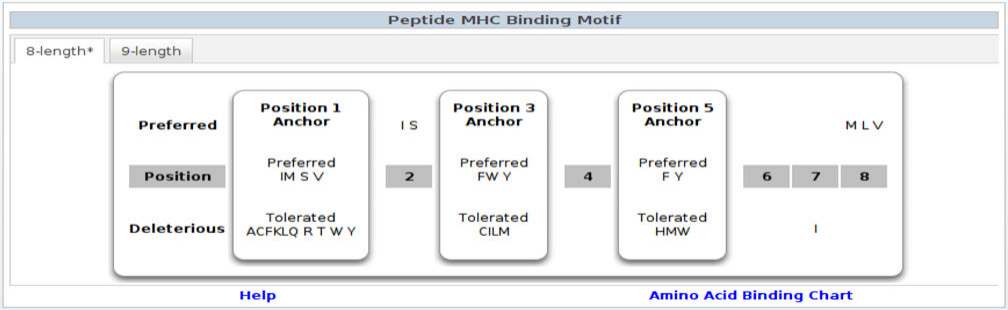
Graphic representation of MHC binding motif for H-2-Kb molecule

**Figure 2b:**
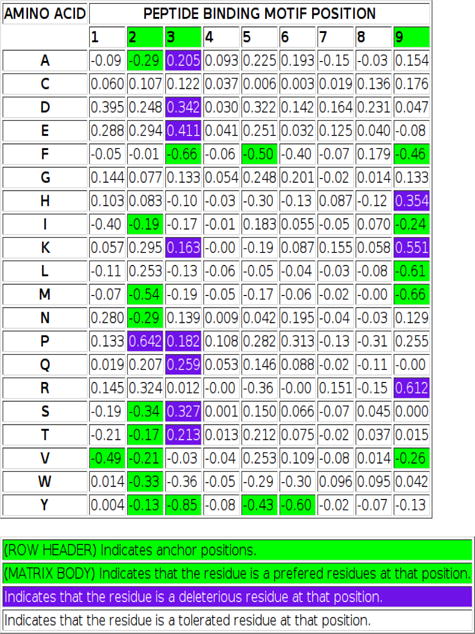
A sample amino acid binding matrix with color-coding to indicate anchor position, and preferred, deleterious, and tolerated residues as a function of position.

**Table 1:** Experimentally measured binding affinities for 18 human and mouse MHC class I alleles used in this study

allele	length	all	binders
H-2-Db	9	1204	263
H-2-Dd	9	201	13
H-2-Kd	9	298	126
H-2-Kk	9	164	78
HLA-A*0201	9	3319	1392
HLA-A*3001	9	641	351
HLA-A*3201	9	573	275
HLA-A*6802	9	2223	562
HLA-B*0702	9	675	211
HLA-B*0801	9	608	297
HLA-B*1501	9	668	388
HLA-B*1503	9	496	348
HLA-B*2705	9	435	155
HLA-B*3501	9	560	155
HLA-B*5101	9	539	83
HLA-B*5301	9	539	161
HLA-B*5401	9	539	91
HLA-B*5801	9	278	66
average	9	776	279
min	9	164	13
max	9	3319	1392

**Table 2:** Confusion matrix shows that automatic algorithm can effectively identify anchor and non-anchor positions based on SMM scoring matrix alone

		Actual	
		Anchor	Non-anchor
**Predicted**	**Anchor**	32	4
	**Non-anchor**	7	119

**Table 3a:** Comparison of anchor residue preference identified with automatic approach with published motifs and those using positional scanning combinatorial libraries

		Pool sequencing motif					Combinatorial library motif					Automatically derived motif				
System	Allele	P2	P3	P5	P6	P9	P2	P3	P5	P6	P9	P2	P3	P5	P6	P9
HLA	A*0201	LM[IVATQ]				VL[MIAT]	L[MQ]				VI[LA]	LM				VL
	A*3001	YF				L		RK			KA[LVIY]		KR			K
	A*3201	[MLITVQS]				[WIFYHT]	TMIQLVS[A]				FIYLW	MLTV				FIW
	A*6802	[LMTVATQS]				[LMTVAT]	VTS[LALMP]				VALI	TV				V
	B*0702	P				L[FWYIVMA]	P[VA]				LFAYI[M]	P				FML
	B*0801		RK	RK		LIVM			RHKF	F[RH]	LFMVIA[E]			HKR	F	FIML
	B*1501	QL				FY					FY[M]	MLQ				FY
	B*1503	QK				FY	QMK[LHASE]				F[MY]	KMQ				FY
	B*2705	R				KRLYANFMIK	R					R.	FLMWY			FIHKMLRY
	B*3501	P				YFMLI[WVA]	PA				FYM[A]	P				FMY
	B*5101	APG				VI[FWYLMA]	PA[GQVS]				I[V]	P				IV
	B*5301	P				WFL[YIVMA]	PA[IY]				FC[IW]	P				W
	B*5401	P				[FWYLIVMA]	A[P]				AV	P				A
	B*5801	AST				FW	STA[VG]				WFIY[MC]	AST				w
H-2	Db			N		LIVM			N[L]		IML[VF]			N		IML
	Dd	G	P	RK		LFI	G	P			FLI[C]	G	P			FIMLRTV
	Kd	YF				LIYM	Y				ILV[M]	Y				HIL
	Kk	E				LIVM	E[D]				IV[FL]	E				I

**Table 3b:** Comparison of anchor residue preference identified with automatic approach with those documented in SYFPEITHI database.

		SYFPEITHI motif					Automatically derived motif				
System	Allele	P2	P3	P5	P6	P9	P2	P3	P5	P6	P9
HLA	A*0201	LM				VL	LM				VL
	A*3001	YF				L		KR			K
	A*3201						MLTV				FIW
	A*6802						TV				V
	B*0702	P				L	P				FML
	B*0801		K						HKR	F	FIML
	B*1501						MLQ				FY
	B*1503	QK				FY	KMQ				FY
	B*2705	R				FL	R	FLMWT			FIHKMLRY
	B*3501	P				YFMLI	P				FMY
	B*5101	APG				VI	P				IV
	B*5301	P				WFL	P				W
	B“5401	P					P				A
	B*5801	AST				FW	AST				w
H-2	Db			N		LIVM			N		IML
	Dd		P			LFI	G	P			FIMLRTV
	Kd	YF				LIV	Y				HIL
	Kk	E				IV	E				I
